# Assessment of insulin resistance in patients with primary hyperparathyroidism before and after Parathyroidectomy

**DOI:** 10.1002/edm2.294

**Published:** 2021-08-05

**Authors:** Soudabeh Nikooei Noghani, Nasrin Milani, Mozhgan Afkhamizadeh, Mona Kabiri, Shokoufeh Bonakdaran, Leila Vazifeh‐Mostaan, Mahdi Asadi, Negar Morovatdar, Masoud Mohebbi

**Affiliations:** ^1^ Department of Internal Medicine Faculty of Medicine Mashhad University of Medical Sciences Mashhad Iran; ^2^ Faculty of Medicine Metabolic Syndrome Research Center Mashhad University of Medical Sciences Mashhad Iran; ^3^ Faculty of Medicine Clinical Research Development Unit Ghaem Hospital Mashhad University of Medical Sciences Mashhad Iran; ^4^ Department of ORL‐Head & Neck Surgery Faculty of Medicine Otolaryngologist – Head & Neck Surgeon Mashhad University of Medical Sciences Mashhad Iran; ^5^ Faculty of Medicine Surgical Oncology Research Center Mashhad University of Medical Sciences Mashhad Iran; ^6^ Faculty of Medicine Clinical Research Development Unit Imam Reza Hospital Mashhad University of Medical Sciences Mashhad Iran

**Keywords:** insulin resistance, parathyroidectomy, primary hyperparathyroidism

## Abstract

**Background:**

Primary hyperparathyroidism (PHPT) can lead to renal and skeletal disorders, as well as insulin resistance and impaired glucose metabolism. The current study aimed to assess the effects of parathyroidectomy on insulin resistance in patients with PHPT.

**Materials and Methods:**

The present study was conducted on 65 patients with PHPT and indications for parathyroidectomy who were referred to the endocrinology clinics of Mashhad University of Medical Sciences. Thereafter, the demographic characteristics of the patients were recorded. Blood tests, including haemoglobin A1c (HbA1c), fasting blood glucose (FBG) and insulin levels, were assessed one week before and three months after the surgery. The insulin resistance score (HOMA‐IR) was calculated and compared using the relevant formula.

**Results:**

A total of 65 participants with a mean age of 45.44 ± 9.59 years were included in the current study. In one‐month postoperative tests, mean scores of FBG (*p *< .05), insulin level (*p *< .05) and HbA1c (*p *< .05) were significantly reduced. Moreover, the HOMA‐IR index decreased in 51 patients after the surgery.

**Conclusion:**

According to our findings, parathyroidectomy can be effective in the reduction of insulin resistance and corresponding complications in patients with PHPT in the present short‐term study. However, it has yet to be confirmed as a treatment method for insulin resistance in these patients. Future long‐term studies are required to be done to investigate the effect of parathyroidectomy on insulin resistance.

## Abstract 2

The present study aimed to assess the effects of parathyroidectomy on insulin resistance in patients with primary hyperparathyroidism (PHPT). The present study was conducted on 65 patients with PHPT and indications for parathyroidectomy. Demographic characteristics of the patients were recorded. Blood tests, including fasting blood sugar (FBS), haemoglobin A1c (HbA1c) and insulin levels, were assessed one week before and three months after the surgery. The Homeostatic Model Assessment for Insulin Resistance (HOMA‐IR index) was calculated. In one‐month postoperative tests, mean scores of FBS (*p *< .05), insulin level (*p *< .05) and HbA1c (*p *< 0.05) were significantly reduced compared to these variables prior to the surgery. Moreover, the HOMA‐IR index decreased in 51 patients after the surgery. As evidenced by the results of the present study, parathyroidectomy can be effective in reducing insulin resistance and its complications in patients with primary hyperparathyroidism (PHPT).

## 1 INTRODUCTION

Evidence has suggested that the risk of impaired glucose tolerance and diabetes mellitus (DM) is higher in patients with primary hyperparathyroidism (PHPT).^1^, ^2^, ^3^, ^4^ Based on clinical observations, the prevalence of diabetes among patients with PHPT is about 8%. In another study, the prevalence of diabetes in these patients was estimated at 15.3%, compared to normal individuals.^2^ Moreover, diabetic patients are up to 1% more likely to develop PHPT.^3^ Patients with PHPT have a threefold increase in the risk of developing diabetes and glucose intolerance, compared to the normal population.^2^, ^3^, ^5^ In these patients, hypophosphataemia, hypercalcaemia and high parathyroid hormone can increase insulin resistance through various mechanisms.^1^, ^6^ In particular, there is a positive relationship between calcium levels and impaired glucose metabolism independent of bone metabolism.^6^

The increase in insulin resistance in these patients can be ascribed to a rise in free intracellular calcium. This calcium, in turn, reduces glucose transport through the insulin‐stimulated glucose transport channel, increasing the need for insulin. If this resistance develops, it can lead to impaired glucose tolerance or diabetes mellitus in the long run.^3^ Despite this association, there is still a heated debate over the role of parathyroidectomy in the improvement of insulin resistance in patients with PHPT.

Some studies suggested that parathyroidectomy in patients with PHPT does not improve glucose metabolism,^5^, ^7^, ^8^, ^9^ while some evidence is against this finding.^10^ Nonetheless, some other investigations indicated that parathyroidectomy can reverse diabetes and impaired glucose tolerance in some patients.^3^

Population‐based studies for evaluating the effect of parathyroidectomy on insulin resistance are limited. Moreover, these studies have shown that diagnosis of insulin resistance based on the optimal cut‐off of the homeostatic model assessment of insulin resistance highly depends on the race.^11^ The present study strived to assess the effect of parathyroidectomy on insulin resistance in patients with PHPT in the Middle Eastern population for the first time. The diagnosis of secondary problems of clinical diseases has been always of utmost importance. Therefore, parathyroidectomy can be considered for addressing the problems of glucose metabolism in people with PHPT. The assessment of the effectiveness of this surgery in the improvement of insulin resistance can be of great help in the reduction of complications and problems caused by insulin resistance in patients with PHPT.

## 2 MATERIALS AND METHODS

### 2.1 Study design

The current study was conducted based on a retrospective cohort design in the endocrine clinic of Imam Reza and Ghaem teaching hospitals affiliated with Mashhad University of Medical Sciences from March 2016 to October 2020. A number of 65 patients with PHPT and indications for parathyroidectomy were selected via the sequential sampling method. Ethical considerations were considered for inclusion in the study. Before the commencement of the study, all patients were provided with the aims and procedure of the study. Moreover, written informed consent was obtained from all of them.

Upon their willingness to withdraw from the study, the patients were removed from the study, and their information was deleted from the database. Patients’ data were recorded considering privacy complete confidentiality. Moreover, the results were reported anonymously. This research project was approved by the Ethics Committee of Mashhad University of Medical Sciences on 3/14/2019 (IR.MUMS.MEDICAL.REC.1398.308). Among the patients referred to the centres, those (within the age range of 16–75 years) with PHPT and GFR (Glomerular Filtration Rate)> 60 were selected for parathyroidectomy. These patients had asymptomatic or symptomatic PHPT (nephrolithiasis, symptomatic hypercalcaemia). Moreover, they had indications for parathyroidectomy according to the Fourth International Workshop guidelines in 2014.^12^ The indication criteria entailed (1) calcium level 1 mg/dL over the upper limits of normal, (2) age of fewer than 50 years, (3) spinal fracture confirmed by magnetic resonance imaging, computed tomography (CT) and vertebral fracture assessment, (4) bone mineral density (BMD) test by dual‐energy X‐ray absorptiometry (DXA): T‐score <–2.5 at the total hip, lumbar spine, distal 1/3 radius or femoral neck, (5) urinary calcium ≥400 mg/24 h, (6) increased stone risk by biochemical stone risk analysis and (7) the presence of kidney stones or nephrocalcinosis confirmed by radiography, ultrasound or CT scan. On the other hand, the exclusion criteria were as follows: diabetes, a history of taking hyperglycaemic medicines, such as thiazide compounds, hyperglycaemia‐inducing diseases (eg acromegaly and Cushing's disease), pregnancy, malignancy and patients who were untreated and had hypercalcaemia after a single parathyroidectomy.

**FIGURE 1:**
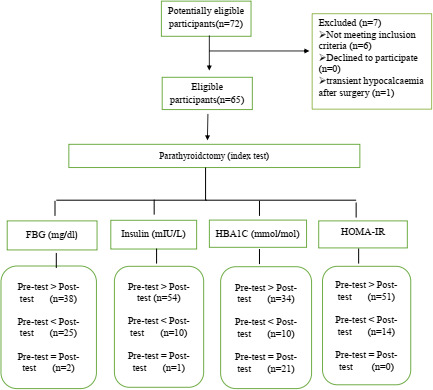
Study design. FBG, fasting blood glucose; HbA1C, haemoglobin A1c; HOMA‐IR, Homeostatic Model Assessment for Insulin Resistance

Thereafter, the following information was assessed one week before the surgery using the designed checklist: (1) the characteristics of the disease, such as age and disease duration, (2) blood test results, including FBG, HbA1C and insulin levels and (3) the information obtained from physical examination of patients, such as body mass index, height and weight. The procedure was performed based on the flow chart illustrated in Figure 1.

Fasting blood glucose (FBG) was checked with the enzymatic colorimetric method using the glucose oxidize test. Serum calcium was measured with the enzymatic method. Serum calcium levels (normal range 8.5–10.4 mg/dL) were adjusted according to the albumin level. Serum parathormone levels (PTH) were measured by chemiluminescence assay. Fasting insulin levels were measured by immunoradiometric method (immunotech kit, Beckman Company) with inter‐assay coefficients of variation (CV) 4.3% and intra‐assay (CV) 3.4%.

Furthermore, the patients with treated hyperparathyroidism who were eligible for the study underwent blood sampling three months after the surgery, and the alterations were assessed. The data were analysed after performing the tests. We should mention that the only patient who experienced transient hypocalcaemia after surgery for 24 h was excluded from the study.

Homeostasis model assessment of insulin resistance (HOMA‐IR) is designed for large epidemiological studies. It is the most commonly used method for the measurement of insulin resistance in vivo and an alternative method to glucose clamp.^13^ The HOMA‐IR index was calculated and compared using the corresponding formula.^13^

$$\text{HOMA}‐\text{IR}:\text{fasting insulin}\;\left(U/L\right)\times \text{fasting glucose}\left(\text{mg}/\text{dL}\right)/405$$

The cut‐offs were used for the homeostasis model assessment of insulin resistance (HOMA‐IR) in the present study. Severe insulin resistance was defined for HOMA‐IR > 3.9. HOMA‐IR was considered to be moderate between 2.5 and 3.9 (2.5 ≥ HOMA‐IR ≥ 3.9), and lack of resistance was considered for HOMA‐IR value <2.5.^13^

### 2.2 Statistical methods

Firstly, the demographic characteristics and other variables of patients were described using descriptive statistics for quantitative variables, as well as frequency and percentage for qualitative variables. Subsequently, the results of analytical statistics were presented. For statistical analyses, the normal distribution of quantitative variables was initially evaluated using the Shapiro‐Wilk test. The mean ± standard deviation or median with percentile 25–75 was reported for the parametric and nonparametric quantitative variables, respectively. The paired sample t test or nonparametric Wilcoxon test was used to compare quantitative variables including the levels of FBG, insulin, HbA1c and HOMA‐IR before and after the surgery. Groups without insulin resistance and the ones with moderate resistance before and after parathyroidectomy were analysed using the McNemar test. Additionally, mentioned groups were compared based on gender by Fisher's exact test. The Pearson correlation was utilized to assess the relationship between variables such as age, BMI and HOMA‐IR index before and after the surgery. The *p*‐value <.05 was considered statistically significant.

## 3 RESULTS

The present study was conducted on 65 cases, and the frequency of females and males was 38 (58.5%) and 27 (41.5%), respectively. The demographic characteristics of the participants are displayed in Table 1.

**TABLE 1:** The mean and standard deviation of quantitative underlying variables in the study population

Variables	Mean	Standard Deviation	Minimum	Maximum
Age (year)	45.44	9.59	25	63
BMI (kg/m^2^)	26.65	2.26	21.20	32.40
Weight (kg)	75.86	9.44	53	99
Height (m)	1.68	0.07	1.55	1.81
Age of onset for the disease (year)	45.44	9.59	25	63

Abbreviation: BMI, body mass index.

According to Table 2, the levels of FBG, insulin, HbA1c and HOMA‐IR variables were compared one week before and three months after parathyroidectomy. There was a significant difference between the test results before and after the surgery in terms of FBG (*p* = .01), insulin (*p* = .0001), HbA1c (*p* = .0001) and HOMA‐IR (*p* = .0001).

**TABLE 2:** Fasting glucose, insulin level, haemoglobin A1C, homeostasis model assessment of insulin resistance, parathyroid hormone, calcium and phosphorus levels before and after parathyroidectomy

Variables	Before parathyroidectomy	After parathyroidectomy	*p*‐Value
FBG (mg/dL) (Mean ± SD)	87.55 ± 7.94	85.83 ± 7.22	.01*
Insulin (pmol/L) (Median; percentile 25–75)	10.4; 8.90–11.90	9.8; 8.20–11.07	.0001**
HbA1C (mmol/mol) (Median; percentile 25–75)	5; 4.65–5.20	5; 4.45–5.10	.0001**
HOMA‐IR (Mean ± SD)	2.21 ± 0.69	2.02 ± 0.64	.0001*
PTH (pg/mL) (Mean ± SD)	112.49 ± 45.95	33.12 ± 9.83	.0001*
Calcium (mg/dL) (Mean ± SD)	11.15 ± 0.39	9.91 ± 0.46	.0001*
Phosphorus (mg/dL) (Mean ± SD)	2.85 ± 0.38	—	—

Abbreviations: FBG, fasting blood glucose; HbA1C, haemoglobin A1c; HOMA‐IR, Homeostatic Model Assessment for Insulin Resistance; PTH, parathyroid hormone; SD, standard deviation.

*Paired *t* test; **Wilcoxon signed‐rank test.

Additionally, the results of PTH, calcium and phosphorous levels are presented in Table 2. The significant differences were indicated for PTH (*p* = .0001) and calcium levels (*p* = .0001) before and after the parathyroidectomy. The level of phosphorous was 2.85 ± 0.38 before the surgery, and we have no access to the phosphorous level of patients after the parathyroidectomy.

It is noteworthy that some patients had moderate insulin resistance (HOMA‐IR > 2.5 and ≤3.9) and some others had no resistance (HOMA‐IR < 2.5). The frequency of patients in each group and their changes before and after the surgery were examined in detail. The comparison of groups without insulin resistance and with moderate insulin resistance is shown in Table 3. The frequency of HOMA‐IR value as an indication of insulin resistance was significantly different (*p* = .031) following the surgery. It is illustrated that the number of patients decreased from 21 to 15 in the moderate insulin resistance group after parathyroidectomy (Table 3). The groups including no insulin resistance and moderate insulin resistance were compared based on gender, and the results are summarized in Table 4. There was no significant difference between groups before (*p* = .791) and after the surgery (*p* = .967).

**TABLE 3:** Comparison of the groups without insulin resistance and with moderate insulin resistance before and after the surgery

Groups	HOMA‐IR before the surgery		HOMA‐IR after the surgery		*p*‐Value
	Number	%	Number	%	
No insulin resistance	44	67.7	50	76.9	
Moderate resistance	21	32.3	15	23.1	.031*
Total	65	100	65	100	

Abbreviation: HOMA‐IR: homeostatic model assessment for insulin resistance.

*McNemar's test.

**TABLE 4:** Comparison of the groups including no insulin resistance and moderate insulin resistance based on gender before and after parathyroidectomy

Gender	HOMA‐IR before the surgery		*p*‐Value	HOMA‐IR after the surgery		*p*‐Value
	No resistance	Moderate resistance		No resistance	Moderate resistance	
Male	19	8		21	6	
Female	25	13	.791*	29	9	.967*
Total	44	21		50	15	

Abbreviation: HOMA‐IR, homeostatic model assessment for insulin resistance.

*Fisher's exact test.

Table 5 demonstrates the results of correlation between various variables including age, BMI and HOMA‐IR. Accordingly, a weak correlation between age and BMI with HOMA‐IR before and HOMA‐IR after the surgery was found, with no significant differences between mentioned variables. Furthermore, there was a strong significant correlation between HOMA‐IR before and HOMA‐IR after parathyroidectomy (*r* = 0.933, *p* = .0001).

**TABLE 5:** Pearson's correlation of different variables including age, BMIand HOMA‐IR before and after the surgery

Variables	Age	BMI	HOMA‐IR Pre‐test	HOMA‐IR Post‐test
Age				
*r*	1	0.176	0.085	0.102
*p*‐Value	—	.161	.502	.420
BMI				
*r*	0.176	1	0.221	0.171
*p*‐Value	.161	—	.076	.174
HOMA‐IR Pre‐test				
*r*	0.085	0.221	1	0.933**
*p*‐Value	.502	.076	—	.0001
HOMA‐IR Post‐test				
*r*	0.102	0.171	0.933**	1
*p*‐Value	.420	.174	.0001	—

Abbreviations: BMI, body mass index; *r*, correlation coefficient.

**Correlation is significant at the 0.01 level.

## 4 DISCUSSION

The present study aimed to evaluate the effect of parathyroidectomy on insulin resistance and FBG in patients with PHPT. The findings of this cohort study revealed that the levels of FBG, insulin, HbA1c and the HOMA‐IR were significantly altered three months after the surgery, compared to one week before the surgery, signifying a marked improvement. Our study suggested that age, gender and BMI were not significantly effective in insulin resistance (HOMA‐IR values) which was previously reported in other studies.^14^ The question is whether glucose control can be an indication for parathyroidectomy in patients with PHPT.

The conducted studies have yielded contradictory results regarding the positive effect of parathyroidectomy on insulin resistance. Some studies have confirmed the role of parathyroidectomy in controlling glucose metabolism^1^, ^10^, ^15^, ^16^ and pointed to its effectiveness in the improvement of insulin resistance.^1^, ^10^, ^15^, ^17^ On the other hand, some others did not approve of this effect^5^, ^7^, ^8^, ^9^, ^18^, ^19^, ^20^ and stated that parathyroidectomy surgical treatment did not improve insulin resistance in patients currently selected for parathyroidectomy.^9^

Some researchers followed up patients for 18 months; nonetheless, they did not observe any changes in glucose metabolism due to parathyroidectomy.^21^ Moreover, some other studies indicated that parathyroidectomy can reduce insulin resistance in some patients but not all of them.^3^, ^6^ The majority of these studies were limited, retrospective and conducted on 9–45 patients. Study participants were followed up at different time intervals ranging from two months to one year. Initial studies started in 1992.^16^ Apart from FBG, risk factors for cardiovascular disease, lipid profile, calcium, phosphorus and vitamin D were checked in some of these studies.^1^, ^7^, ^9^

In a post‐parathyroidectomy study on patients with PHPT, adiponectin levels were clearly elevated despite no changes in lipid profile and insulin resistance.^7^ The studies that did not find any association between insulin resistance and parathyroidectomy observed an improvement in insulin resistance index in people with a higher insulin resistance index (HOMA‐IR > 1.2) after parathyroidectomy, compared to those with HOMA‐IR < 1.^9^ Finally, endocrinologists pointed to improvement and control of glucose in 77% of PHPT patients with type I or type II diabetes mellitus.

Therefore, impaired glucose metabolism can be considered an indication for surgery in this group of patients.^5^ In this regard, the results of a related study demonstrated that fasting glucose (5.6 ± 1 to 5.4 ± 0.8 mmol/L) and two hours after a meal (7.2 ± 3 to 6.3 ± 3.1 mmol /L) decreased in patients with PHPT after parathyroidectomy. Moreover, 50% and 33% reductions were observed in DM frequency and pre‐diabetes, respectively.^10^

The mechanism through which parathyroidectomy can reduce insulin resistance is still a debated issue. Various factors were assessed to unveil this relationship. It has been hypothesized that serum levels of calcium, phosphorus and PTH can be effective on insulin resistance.^3^ Phosphorus plays an important role in energy production, and low levels of phosphorus can lead to impaired energy metabolism and insulin resistance.^22^ In a study, phosphorus levels were inversely related to glucose two hours after a meal and positively correlated with insulin resistance.^23^ Low levels of serum phosphorous have also led to increased insulin resistance and impaired fasting glucose.^22^ Recent studies also have shown that lower serum phosphate and higher levels of calcium are associated with increased insulin resistance in healthy individuals.^24^

For instance, in a study, phosphorus levels were inversely related to glucose two hours after a meal and positively correlated with insulin resistance.^23^ Recent studies also have shown that lower serum phosphate and higher levels of calcium are associated with increased insulin resistance in healthy individuals.^24^ Particularly, low levels of phosphorous can lead to increased insulin resistance and impaired fasting glucose.^22^ Phosphorus plays an important role in energy production, and low levels of phosphorus can lead to impaired energy metabolism and insulin resistance.

Another important factor that should be considered in this regard is vitamin D. Recent evidence has shown that vitamin D deficiency is effective in the pathogenesis of the metabolic syndrome. The role of vitamin D in insulin secretion and improving insulin resistance has been demonstrated.^24^ The parathyroid hormone in the kidney increases the synthesis of 1,25 OH vitamin D in the proximal tubule. On the other hand, Vitamin D deficiency or insufficient levels of Vitamin D are very common in patients with primary hyperparathyroidism. Vitamin D deficiency and hyperparathyroidism can lead to a decreased calcium levels in serum compared to the normal range.^25^ Furthermore, vitamin D deficiency reduces calcium level in peripheral tissues leading to a reduction in insulin secretion from the beta cells of pancreatic and subsequently increases insulin resistance in obese patients.^26^

In another study, 21 patients with symptoms of PHPT underwent parathyroidectomy. Fasting blood glucose, calcium, phosphorus, plasma insulin and vitamin D were measured at baseline and two months after parathyroidectomy. According to the results of the stated study, the mean scores of insulin resistance (HOMA‐IR) were obtained at 2.21 ± 0.69 and 2.02 ± 0.64 at the commencement of the study and two months after the surgery, respectively, pointing to a significant decrease (*p* = .0001). Moreover, phosphorus levels increased, and no changes were observed in vitamin D and glucose levels.^1^

Along the same lines, another study suggested that calcium levels were associated with impaired glucose metabolism independent of PTH and bone metabolism. The results of the referred study did not find a positive relationship between PTH and insulin resistance. This relationship remained significant after adjustment for PTH, age, body weight, height, phosphorus composition of bone markers and kidney function.^6^

As mentioned earlier, PHPT is a relatively common disease. The complications of this disease highlight the need for further studies to better understand this disease, treatment methods and the related factors. Information about the effect of parathyroidectomy on insulin resistance in patients with PHPT can form the basis of planning to minimize the complications of this disease.

### 4.1 Limitations, strengths and weaknesses

Regarding the limitations of the present study, one can refer to the duration of the study and patient follow‐up. Participants were followed up for three months after the surgery. No information is available on long‐term follow‐up and the positive effect of parathyroidectomy in the long term. Due to unforeseen circumstances during the pandemic, we were unable to evaluate the weight changes of the participants after parathyroidectomy. In this study, we had to use the homeostasis model assessment of insulin resistance (HOMA‐IR) as a result of limited resources. We believe that using the euglyacaemic hyperinsulinaemic clamp technique could result in a more accurate insulin assessment. The euglycaemic hyperinsulinaemic clamp would have been ideal for studying insulin.

The role of vitamin D as a potential confounder was not determined in this study due to costs associated with measurements of vitamin D in our hospital settings. However, we did not expect a major change in vitamin D levels within 3 months based on guidelines and former studies. Patients in our study had indications for parathyroidectomy according to the Fourth International Workshop guidelines in 2014.^12^ The level of vitamin D was not included in the criteria of the guideline. In addition, according to previous studies, after parathyroidectomy in patients with PHPT, despite the increase in the basic level of vitamin D, the level of the vitamin may remain deficient or insufficient.^27^

On the other hand, one of the notable strengths of the present study was assessing the role of parathyroidectomy in the reduction of insulin resistance for the first time in the Iranian population. Another strength was the elimination of individuals with decreased kidney function (GFR < 60). Since kidney failure can lead to gluconeogenesis and affect insulin resistance, it can be a contributing factor to increased PTH.

### 4.2 Suggestions for further research

It is recommended that future studies be performed on a larger sample size in several different geographical areas and races. Moreover, it is suggested that participants be followed up for a longer time. Besides, in future studies, the severity of hypercalcaemia also needs to be considered.

## 5 CONCLUSION

Based on previously conducted studies, PHPT can lead to insulin resistance (the hormone that regulates blood sugar levels) and impaired glucose metabolism. Nevertheless, there are conflicting results on improved insulin resistance in patients with primary hyperthyroidism after parathyroidectomy (surgical removal of one or more parathyroid glands). In the present study, there was a significant difference between the test results one week before the surgery and three months postoperatively in terms of FBG, insulin, HbA1c and HOMA‐IR. According to the laboratory data, parathyroidectomy can be effective in the prevention of insulin resistance and its complications in patients with PHPT. Although a considerable change was observed in the insulin resistance, FBG and HbA1c for studied patients following the surgery, parathyroidectomy has not been proven to be the definitive treatment of insulin resistance in these selected patients. Longer follow‐up studies are required to study the effect of parathyroidectomy on insulin resistance.

## CONFLICTS OF INTEREST

The authors declare that they have no conflict of interest regarding the publication of the current article.

## AUTHOR CONTRIBUTIONS

SN, MM, MA and NM were primary investigators responsible for the study design, data collection and manuscript preparation. SB, MAf, MAs and NM were responsible for the critical revision of important intellectual contents. MK and NMo were statistical consultants contributing to data analysis. MM, NM, MK and SN contributed to some parts of the manuscript preparation and critical revision of important intellectual content.

## Data Availability

The data that support the findings of this study are available on request from the corresponding author. The data are not publicly available due to privacy or ethical restrictions.
